# Complexity in Health: Can Design Help Support Interdisciplinary Solutions?

**DOI:** 10.9745/GHSP-D-21-00222

**Published:** 2021-11-29

**Authors:** Ledia Andrawes, Tracy Johnson, Michael Coleman

**Affiliations:** a Sonder Collective, London, United Kingdom.; b Bill & Melinda Gates Foundation, Seattle, WA, USA.; c Common Thread, Dublin, Ireland.

## Abstract

Public health challenges are increasingly complex and won’t be solved through traditional methods by the public health community alone. Design, with its people-centered approach and collaborative practice to harness a diversity of perspectives, can facilitate interdisciplinary efforts to creatively resolve tough global health challenges.

## Key Messages

- The collaborative nature of design practices is well-suited to creating the conditions for productively integrating varied disciplines to solve challenging problems in global health.
- The tensions that can arise from merging different disciplines and approaches within public health and beyond, rather than hinder progress, can be surfaced for stronger solutions to emerge.
- We explore 3 productive tensions that result from integrating global health and design:
  1. Integrating explicit and implicit knowledge
  2. Challenging linearity with iteration
  3. Enabling collective ownership of processes and solutions
- For these tensions to be productive, design and global health practitioners must do more to be open and adapt to the heritage of other disciplines; both those that have always played a role in global health and those that are just beginning to garner attention in the field.
- Designers can do more to establish early that they are not there to replace but rather to “accompany” other disciplines through collaboration.

## INTRODUCTION

Global public health programming has become increasingly complex. Overlapping investments aim at developing health policy, extending the reach of supply chains, supporting more effective service delivery, and addressing demand-related barriers to improve health. Whether it’s vaccine hesitancy, antimicrobial resistance, or health care worker motivation, there is an increasing recognition that many of the problems facing the global health field have human behavioral dimensions that are often poorly understood or addressed. While this recognition has driven many global health program strategists to increasingly integrate qualitative and participatory approaches in program design as well as mixed methods for evaluation, a premium continues to be placed on global health professionals with substantive expertise—often biomedical and heavily quantitative—over the experiential and contextual insights that emerge from direct engagement with end users.

There is general agreement that the global coronavirus disease (COVID-19) pandemic has exposed and accentuated entrenched social inequities, revealing once again how vulnerable population groups—whether based on gender, disability, age, ethnicity, or geography, among others—are disproportionately affected.^1^ The Bill & Melinda Gates Foundation’s 2020 Goalkeepers Report summarized this concept well^2^:

*In the blink of an eye, a health crisis became an economic crisis, a food crisis, a housing crisis, a political crisis. Everything collided with everything else.*

Health is contextual—social, cultural, and behavioral—and the difficulties of taking into account more upstream systemic and social considerations have required global health programming to focus downstream, on individual behavior change and individual drivers of seeking, adopting, and adhering to treatment.^3^

The potential value of integrating varied disciplines to bring new insights and solutions to complex challenges is well-accepted in principle in global health. We argue in this commentary that more can be done. Drawing on the concepts and expertise of different disciplines does not automatically make a project interdisciplinary. True **interdisciplinarity** involves integrating information, concepts, tools, and rules that are used or produced by different disciplines on a particular subject. One might think about it less as forming a band and more as forming an orchestra where the musicians trade instruments. Yet, as we discuss in more detail, greater clarity on what the effective integration of disciplines looks like is needed. A clear and replicable process to support exactly how disciplinary boundaries can be minimized would help teams to identify unique solutions necessary for engaging the social, political, economic, and behavioral foundations that determine population health.^4^^–^^6^ Interdisciplinary practice is hard work, demanding “constant explanation, adaptation and scientific readjustment” from all practitioners involved—from those engaged in project planning to research and to problem solving.^7^

Design practices are inherently collaborative. The process, mindset, and approach put end users and their context at the center. Design practices encourage shared understanding among diverse areas of expertise and experience. Individuals, communities, and organizations are active partners in the design and implementation of solutions. This collaborative focus of design can strengthen interdisciplinary ways of working in complex settings by creating the conditions for multiple voices to be heard, considered, and effectively integrated into problem-solving approaches. For the value of design to be optimized, designers can do more to establish early that they are not there to replace but rather to accompany other disciplines through collaboration. In this commentary, we argue that design can create a neutral space and provide a proven process for interdisciplinarity in global public health.

The collaborative focus of design can strengthen interdisciplinary ways of working in complex settings by creating the conditions for multiple voices to be heard, considered, and effectively integrated into problem-solving approaches.

## MAKING THE CASE FOR INTERDISCIPLINARITY

*This landscape in all its beauty, sometimes gentle, sometimes terrible, cannot be seen fully by any one of the occupants of the room. Indeed, it cannot be known fully by a whole generation of men [and women]. Explorers of each generation travel into its unknown recesses and, with luck, return to share their discoveries with us. So the life of the new room would go on—thought, reflection, contemplation—as the explorers bring back their discoveries to share with the room’s occupants. This landscape that we gaze on and try to understand is an epic portion of the human experience.* —Mead^8^

Mead’s plea for a “new room” in that “vast and rambling” house called science reminds us that the landscape of inquiry and problem solving cannot be seen fully by any one discipline. Recognizing that complex problems are not so neatly organized within disciplinary demarcations, the field of global health has sought to work in more interdisciplinary ways throughout its history, although not without some challenges. Embracing interdisciplinarity requires a shift among practitioners to overcome disciplinary specificities, including temporal conflicts in data collection and analysis, the requirements of institutional and disciplinary affiliations, and contrasting theoretical frameworks and methodologies. When successfully overcome, experts can bridge disciplinary divides, propel the collective effort to address the multifactorial drivers of health problems, and together identify new levers for change. Moreover, the unique knowledge that emerges from the dissolution of disciplinary boundaries is essential for addressing imperatives such as equity in human health.

Interdisciplinary training in medical schools, for example, is increasingly encouraged for specialists to consider the needs of patients more holistically. An overly specialized approach can miss valuable insight from the connections between symptoms.^9^ In global health, it is also common for specialists to come together to share expertise, knowledge, and skills to positively impact individual and societal health outcomes.

The case for interdisciplinary teamwork is to enable the integration of knowledge that supports a more thorough understanding of the whole picture, what Marilyn Stember calls the “holistic complex of interrelationships.”^10^ There is no doubt that specialization along with widening the aperture of what is considered expertise has brought great strides in advancing the field of global health. Yet when navigating some complex problems, it demands not only harnessing diverse skills and knowledge but also blending disciplinary boundaries toward a common goal.

As the drive for specialists to come together to impact individual and societal health outcomes grows, so too have the range of terms used to characterize collaborative working arrangements between practitioners from different disciplines. Terms such as intradisciplinary, multidisciplinary, crossdisciplinary, interdisciplinary, and transdisciplinary are used to refer to both different types of teams and different processes within them (Figure). Yet, these terms are often unclear as to how they distinguish between different degrees of collaboration and knowledge integration.

**FIGURE:**
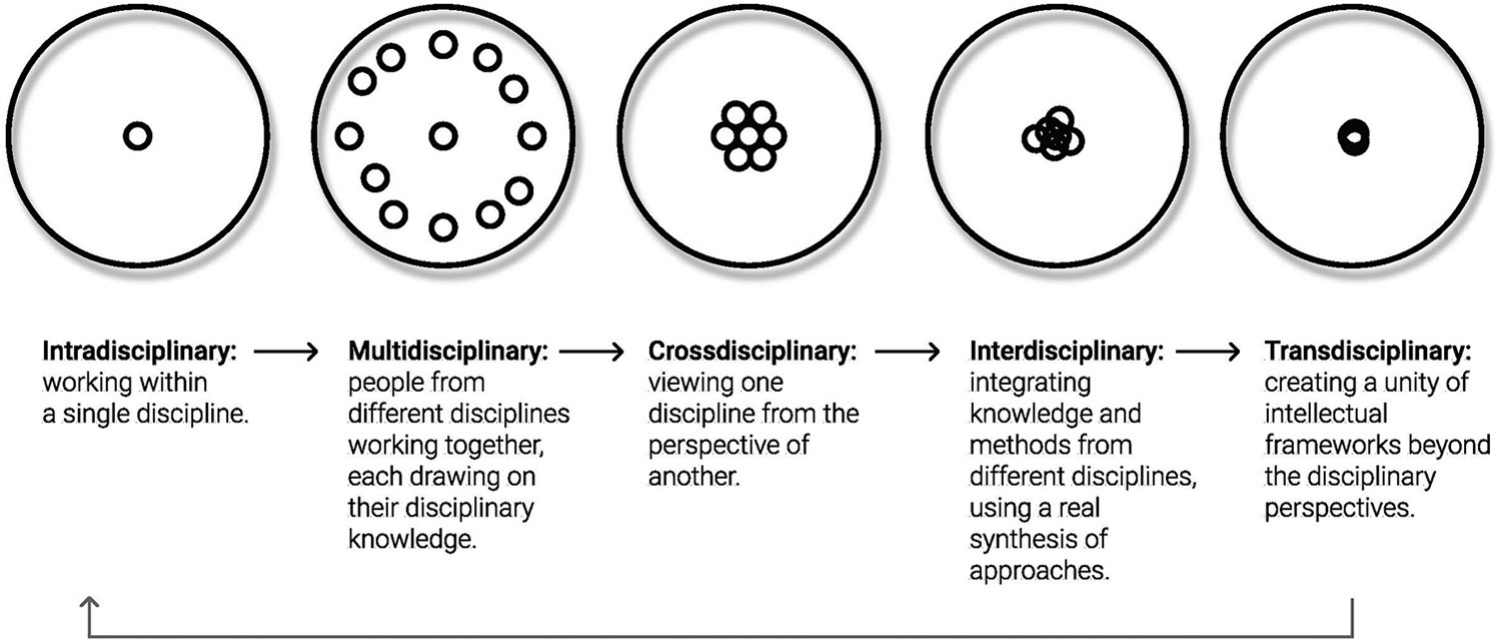
Degrees of Knowledge Integration Within or Across Disciplines^a^ ^a^ Adapted from Stember.^10^

Stember offers an overview of the different degrees of collaboration and knowledge integration within or across disciplines.^10^ Along the spectrum of intra- to transdisciplinary, one can see the range: from **maintaining** intellectual frameworks from a single discipline, to **considering** other disciplinary points of view, to **collaborating** with different disciplines, to **integrating and synthesizing** knowledge from other disciplines, and finally to **unifying** intellectual frameworks beyond disciplinary boundaries.

Given the number of terms to describe this process of working together, it is understandable that as practitioners, we are not always able to fully reflect on the processes to achieve such collaboration. However, simply drawing on the concepts of different disciplines does not automatically make a project interdisciplinary. Going beyond just assembling different types of knowledge, interdisciplinarity is a critical stance in the effort to produce a whole that is greater than the sum of the parts. With interdisciplinarity, practitioners seek out the complexity of interrelationships and integrate the contributions of other disciplines into their own. They do this in ways that dissolve traditional boundaries and open space for new insights and solutions to emerge.^11^

Simply drawing on the concepts of different disciplines does not automatically make a project interdisciplinary.

Interdisciplinarity within global health projects has been questioned before.^5^ On the far end of the spectrum is what some have termed “best-practicitis”^12^—a top-down, “plan and control” approach that more immediately responds to the needs of aid organizations. The argument here is that the development system relies more heavily on practitioners looking for the single right answer rather than a set of diverse solutions, spending more time trying to do things right than determining if they are doing the right things, and creating “how-to” guides and off-the-shelf toolkits that take precedence over attempts to change ways of working through deeper interaction and dialogue.

One does not need to embrace the concept of “best-practicitis” to agree that the global development system has struggled to incorporate more adaptive and integrative approaches, and in so doing has unintentionally reinforced the traditional linearity of problem definition, solution identification, implementation, and evaluation.^12^ Heterogeneous forms of evidence present methodological challenges that perhaps unintentionally reduce willingness to use experiential and relational methods.^13^ More established definitions of success have predominantly fixated on speed, scale, costs, and other critical quantifiable indicators such as intervention coverage and mortality. While these are essential features that the global health field rightly relies upon to ensure that the appropriate standards of quality, safety, and do no harm are met, this approach has conditioned many practitioners to adopt quantitative-heavy practices from their technical areas of expertise, which then perpetuate top-down cultures of procedural quantification and reporting.^14^ This model of impact is not well-suited to accounting for how people’s experiences of the world and their health shape their knowledge and behavior. Design processes that support the co-construction of knowledge can complement traditional processes with the addition of more holistic and diverse forms of evidence. This complementarity can support practitioners to navigate global health challenges that require a deeper understanding of how to design interventions that take into greater account the complexity of human behavior.

## DESIGN AS A FACILITATOR OF INTERDISCIPLINARITY

There is general agreement that interdisciplinarity practices can add value to the complex challenges facing global public health.^4^^,^^5^ Yet, as Kivits et al. ask, how do we do this?^7^

We contend that design is well-suited to creating the conditions for multiple voices to be heard, considered, and more fully integrated when problem solving in interdisciplinary teams. The generative nature of design practices brings a unique form for problem solving. In the last few decades, the design field has expanded its role from shaping a product for a human to shaping relationships between humans in a system.^15^ This role increasingly includes interdisciplinary inquiry that tackles complex sociocultural challenges.^14^

Design is well-suited to creating the conditions for multiple voices to be heard, considered, and more fully integrated when problem solving in interdisciplinary teams.

Design practices can offer experiential and relational ways for looking, listening, sharing, and learning. The collaborative nature of design practices can provide strong guide rails for effectively bringing varied disciplines together to solve problems at different levels of magnification, focus, and interpretation—from the historical context to social norms and right down to the decision point. In settings where various disciplines come together for the common purpose of improving health, design processes reconceptualize social needs by illuminating the why and the how of human behavior and offering collaborative spaces for imaginative solutions.^16^ As many of the articles in this Supplement issue discuss, designers are increasingly expected to operate in ways that bring in more systems thinking, visualize problems and make conceptual ideas concrete, gather and mediate diverse stakeholders, and give prominence to the voice of the people behind particular global health challenges.^14^ By supporting action that starts from people’s agency and relationships, design practices can offer a more human-centered practice for interdisciplinary working.

The need to articulate design’s place among technical experts was foreseen by Richard Buchanan as he addressed the Design Educators Forum of South Africa in 2000^17^:

*Our bigger challenge is to explain why design is different from other "subject-based" disciplines, how it integrates knowledge from many other disciplines, and how it turns theoretical understanding in other disciplines into valuable products that can have great impact on society.*

Now, 20 years later, the need for design to distinguish and establish itself alongside subject-based disciplines that he highlighted has yet to be addressed. This articulation is needed if design is to effectively contribute to global health by creating space and a process for knowledge integration from many disciplines.

Design efforts in the international development, global health, and social innovation spaces have had a long history of embracing multiple disciplines in their practice. Bannon emphasizes that design research and practice have evolved thanks to influences from human factors and human-computer interaction, the methodological contributions of anthropologists and sociologists, organizational information systems research, user-centered design, participatory design, and the more craft-oriented design professions.^18^ Design efforts focused on social innovation,^19^ international development,^20^^–^^22^ and global health^23^^,^^24^ continue to push the potential for interdisciplinary practice in new directions.

These trends shed some light on why a growing number of institutions have either sought out partners to contribute design expertise or have started to build internal design capacity of their own.^25^ Design practices are supporting the shift toward working cultures that allow for integration across the many disciplines devoted to an understanding of human behavior and human values.^26^ For some, integration comes with risks, such as the risk of design practices being reduced to a toolkit approach that can lead to known or obvious conclusions or overweighting individual views at the risk of alienating others.^27^^–^^30^ Furthermore, some design practices have been critiqued for not routinely adhering to ethical research requirements to protect research subjects and their data, which are common practices in the social sciences.^28^

The contrasting methodologies of different disciplines highlight tensions between their respective ways of working. However, rather than see these tensions as impediments, some design practices can treat them as fertile ground to push beyond institutionalized expectations of a solution and to open a space for new opportunities that can lead to creative solutions that are more suited to addressing the messy complexity of people’s expectations, behaviors, needs, and wants related to their health. Bazzano et al. state the^31^:

*central tenets of design thinking research, like iteration, tolerance for ambiguity, pivots, and rapid prototyping, are inherently at odds with some prevailing processes in health and biomedicine, particularly public health, where hypothesis-driven research is the norm.*

Some design practices can treat tensions that arise between disciplines as fertile ground to push beyond institutionalized expectations of a solution.

Design practices can foster greater interdisciplinarity by harnessing such tensions. We explore 3 productive tensions that result from integrating global health and design: (1) integrating explicit and implicit knowledge, (2) challenging linearity with iteration, and (3) enabling collective ownership of processes and solutions.

## HARNESSING PRODUCTIVE TENSIONS BETWEEN DESIGN AND GLOBAL HEALTH

### Productive Tension 1: Integrating Explicit and Implicit Knowledge

In many disciplines, knowledge is often seen as taking an explicit form: things that are written down, defined, categorized, systematized, or quantified. In contrast, knowledge in design is often seen as tacit and implicit: rather than something to be articulated, knowledge exists as embodied in people’s behaviors. Design practices seek to challenge the argument that knowledge **only** counts when it is objective, scientific, statistically valid, or considered “best-practice.” It broadens what constitutes knowledge, including the experience of end users and others. Knowledge that emerges from research and observation generated through standardized (mainly quantitative) methods tends to be valued over other types of knowledge that uses qualitative, participatory, and observational techniques that by nature can be adapted as the research questions evolve. This can create tension with design practices that tend to prioritize relational and tacit types of knowledge derived more directly from the end users’ points of view and which consider this type of knowledge a necessary component in addressing public health challenges.

Design practices seek to challenge the argument that knowledge only counts when it is objective, scientific, statistically valid, or considered “best-practice.”

In a project to redesign the strategy for national public health insurance in Kenya, a design-led approach was chosen by the World Bank Group and the Kenyan Government’s National Hospital Insurance Fund (NHIF) to determine how NHIF could better meet the needs and preferences of the informally employed to achieve greater health insurance uptake and retention. During the 5-month design process, more than 124 people were involved, including 84 citizens, 22 NHIF managers and staff, and 18 stakeholders from health service delivery organizations. The interdisciplinary team comprised 2 designers, 1 sociologist, 1 health economist, 1 marketing specialist, and 1 community mobilizer.^*^

Initially, the various actors involved “labeled” the citizens involved in the project differently.

- The project’s funder labeled them **recipients or beneficiaries.**
- The NHIF and the marketing specialist on the team considered them **customers**.
- The sociologist perceived them as **research informants.**
- The designers saw them as **service-users** and **co-designers.**

As the project progressed, the designers challenged the role of citizens as passive recipients or informants in the eyes of others by inviting citizens to join design workshops as codesigners of possible future scenarios alongside policy makers, NHIF implementers, and the interdisciplinary project team. One of the global health specialists responsible for funding the project shared how design practices^24^:

*…highlighted the issues from a lived experience perspective and codified the project in the language used by real people.* —Participant working for a funding agency

The more participatory emphasis of the design process led the way for a diverse team of project stakeholders to ascribe greater value to the citizens’ tacit knowledge and lived experience. In this case, design succeeded in helping the global health specialist reach her particular goal^24^:

*What I’ve been trying to push is how do we understand users even before we get into defining what the problem is… [this organization] is no different to other places in this regard, we’ve made a decision ahead of time, what are the problems.* —Participant working for a funding agency

The more participatory emphasis of the design process led the way for a diverse team of project stakeholders to ascribe greater value to the citizens’ tacit knowledge and lived experience.

Unfortunately, there was no formal evaluation of whether the solutions proposed through this project increased access to insurance by the informal sector. However, the participants from the World Bank and NHIF senior managers credited the design-led process that guided the work to change the minds of internal NHIF stakeholders on what they needed to “solve for” as an organization and to foster more interdisciplinary collaboration among previously competitive project teams.

This expansion of what constituted knowledge went beyond traditional disciplinary boundaries such that rather than starting with a technical solution, design practices helped ensure project parameters were grounded in a deeper understanding of how people’s experiences shape their consciousness and drive their health-related behaviors. However, this process can invite ambiguity into projects, and forcing people to engage with more ambiguity than they are used to creates tension. Asking experts to re-examine, reconsider, and blend their knowledge with that of others requires trust and a willingness to explore a problem anew. Specialists who have been working in their space for many years can feel their expertise is being shifted from the center to the periphery.

Experience-based and visual design practices can situate the knowledge of users and collaborators in dynamic ways for others to consider again. Visual design practices can bring clarity to diverse teams on otherwise complex and unfamiliar concepts. By turning complex information into sketches, models, and interactive role-plays, such design artifacts embody knowledge that is not as easily communicated using tables, words, and numbers. Many global health practitioners often stop at this point, seeing the role of design to fulfill that communication function. But design’s value comes in its ability to challenge assumptions based on technical expertise alone and instead to create opportunities for explicit knowledge to blend with user perspectives and other types of tacit knowledge. Design practices can further support practitioners in acknowledging where their knowledge sits in relation to others and facilitating teams to collectively move beyond any unrecognized biases.^32^^,^^33^ Resituating a type of knowledge in relation to others enables practitioners to build on the tradition of participatory methods in global health and relate to beneficiaries less as subjects of inquiry and more as collaborators in the desired change.

### Productive Tension 2: Challenging Linearity With Iteration

Many approaches suggested for effectively navigating complex problems contradict what can be a fairly rigid and linear process of problem definition, solution identification, implementation, and evaluation.^12^^,^^34^ While such linear processes can be efficient, they have proven less effective in complex situations where problem definition remains an ongoing, open, and critical reflection throughout the project, rather than just an upfront phase. In contrast to a linear-thinking, single-solution approach born of analysis, an iterative design approach is flexible in nature, continuously skeptical as to the definition of the problem itself, opportunistic in its generation of solutions, and almost obsessed with introducing creative options and experimentation. Such an approach challenges the notion that there is 1 pathway to change. Instead, it acknowledges that dynamic problem solving involves regular cycles of learning and experimentation to reach a solution.^35^ Given the pace of funding cycles that drive for more rapid solution development, a tension arises with design practices that can insist on longer processes of iteratively working through the complexity of the problems.^†^

An iterative design approach challenges the notion that there is one pathway to change.

For people who seek the certainty of externally structured, well-defined problems, iterative design processes have the potential to create discomfort for people who are not used to them. Take, for example, a 5-day design sprint/workshop in Zimbabwe that aimed to use mixed-methods, segmentation-based insights to generate innovative ideas and early prototypes to address poor uptake of voluntary medical male circumcision (VMMC) services. There were approximately 40 people in the sprint/workshop, including policy representatives from the Ministry of Health, practitioners from partner organizations with various technical and programmatic backgrounds, and current and potential clients of VMMC services.

One of the project sponsors reflected afterward on how challenging it was for her to let go of control. This was particularly the case with **unproven** activities the design team used to push participants into a more creative space when generating ideas. For example, an activity provided participants with several rounds of unrealistic scenario prompts that started with “what if…” or “imagine if…” for them to generate more novel ideas. The different ideas were rotated around the room, and participants were invited to build upon those that were generated by others. These less “evidence-based” methods and more creative scenario-based methods to generate additional ideas were difficult for this project implementer to accept on day 2. By day 5, the implementer declared the week as a significant success as it pushed teams to think creatively, work in more interdisciplinary ways, and develop new prototypes that could be further tested and developed.

At the same time, there were moments during the week that the project implementer felt nervous and questioned whether bringing in a design team was a mistake. This implementer shared with an author^24^:

*The approach is the approach, I still can’t cope with the chaos part of it, but the approach is the approach.* —Implementer involved in the project

This discomfort usually occurs when diverse teams are forced to engage with the ambiguity, disorder, and messiness of integrating each other’s knowledge and ideas. Design practices can help global health practitioners navigate ambiguity and open deliberative and adaptive spaces for new possibilities to emerge.^36^ This occurs through experiential and speculative processes, such as ideation and prototyping, that support interdisciplinary teams in maintaining the “parallel lines of thought” necessary for integrating analytical and creative perspectives^37^ and the “double vision” that helps a designer be both learner and creator.^38^

Design practices can help global health practitioners navigate ambiguity and open deliberative and adaptive spaces for new possibilities to emerge.

This movement between the different activities associated with learning, analyzing, and doing are critical characteristics of design when seeking interdisciplinary solutions because answering the wrong question or answering the right question poorly is increasingly costly in complex settings.^39^ However, designers need to negotiate a balance between practices that are human-centered and messy and practices that are predictable, bounded, and meet the institutional and project needs. Despite the aspirations and expectations of some, design practices do not always offer implementers immediate answers to complex problems. Sometimes, as they did in this situation, they provide new and welcome ways of collaboratively navigating intractable problems that persist with no obvious solutions.

### Productive Tension 3: Enabling Collective Ownership of Processes and Solutions

Many global health practitioners are inspired by the sense of possibility that emerges when a community adopts a solution as their own. The global Ebola virus disease and COVID-19 pandemics have demonstrated that to achieve impact and scale solutions that are sustainable over time, global health practitioners must design solutions **with** local communities and not simply **for** them. What constitutes collective ownership can be difficult to clearly define within projects that involve diverse stakeholders with varied agendas. At the same time, there is a growing consensus that enabling collective ownership^40^:

*…demands genuine interactions, creating enabling conditions and spaces for incremental changes, and building shared values.*

The collaborative nature of design practices supports interdisciplinary work by creating spaces that call for the exchange of values-based ideas and nurture a greater sense of collective ownership. Designers can provide a structure for continuous interdisciplinary collaboration by (1) bringing in dialogue-based design methods, (2) stimulating the creativity and ideas of collaborators, and (3) enabling collaborators to bring in their material and intellectual culture.^41^ This suggests that practicing design in interdisciplinary settings can require additional skillsets as designers are also required to be fluent in balancing multiple participant ideas^42^^,^^43^; addressing “changing roles of power” in groups^44^; and developing contextually adapted methods for diverse participants to contribute throughout a process.^45^ Design practices that seek to integrate multiple (and sometimes contradictory) viewpoints that are centered around the end user experience can come into tension with a more multidisciplinary approach in global health—an approach that often includes more perspectives but can unknowingly maintain disciplinary boundaries and undervalue user experience.

In the previously mentioned project seeking to redesign a citizen-centered public health insurance service in Kenya, senior NHIF managers, who would eventually implement the service, determined that collective ownership was critical to the project’s success. Through a series of ethnographic activities and design workshops, stakeholders across different organizational departments, external providers, and citizens from different regions came together to provide unique insights into current challenges and future possibilities associated with such a service. Over time, a culture of reciprocity and knowledge exchange developed, which ultimately created a sense of co-ownership in the final service. One of the key project sponsors reflected on their experience with the design process^24^:

*I found that in this way you are able to involve all the stakeholders, and you look at the situation from all the angles… for me, that was the key thing.* —Implementer perspective

The strategy that was developed was holistic in that it did not only provide the required changes in communications and messaging toward the informally employed but also covered a more holistic set of required changes. These ranged from institutional reforms for improved service quality by health care providers and the need for new financial models to support more vulnerable groups. Although it was design practices that helped to build bridges and collective ownership across different stakeholder groups, it was the commitment of individual stakeholders that was foundational to the implementation of the strategy’s recommendations.

## CONCLUSION

This commentary is an invitation to both designers and public health professionals to join forces more openly and more often to bring together the plurality of expertise within public health and the practical, people-centered, problem-solving approaches of design. For design to genuinely harness interdisciplinary solutions, it requires that practitioners of both design and global health reflect on their respective contributions to the bounded nature of global health programs.

Design will not solve all the problems we are grappling with as a global health community. Where design can contribute is with its convening power and ability to productively bring interdisciplinary teams together toward a common goal. As the articles in this Supplement issue demonstrate, when done well, design can create space for the blending of ideas untethered to narrow communities of practice, academic process, pressures, and tradition. This is an exciting opportunity to expand our definition of knowledge, embrace iteration, and foster collective ownership. Design practices can provide a people-centered framework to make the most of the diverse disciplines and expertise within public health so that they are better able to flourish collectively, creatively, and productively, such that true interdisciplinarity can be harnessed to tackle the toughest global health challenges we face.

## Footnotes

* The team undertook exploratory design research, which received ethics approval from the Africa Medical Research Foundation (AMREF) ethics and scientific review committee (approval number ESRC P168/2015).

^†^
Plan-Do-Study-Act and adaptive management approaches are attempting to bring similar iterative working styles into global health practice. These efforts are emergent and perhaps would be a fruitful avenue for collaboration with design.

## Notes

**Peer Reviewed**
